# Patient Blood Management: a systematic review of current international guidelines

**DOI:** 10.1016/j.bja.2026.03.055

**Published:** 2026-05-12

**Authors:** Daniel Dreher, Gabriel Honnef, Philipp Zoidl, Daniel Auinger, Lioba Heuschneider, Paul Zajic, Martin Rief, Helmar Bornemann-Cimenti

**Affiliations:** 1 Medical University Graz, Graz, Austria; 2 Department of Anaesthesiology and Intensive Care Medicine, Medical University Graz, Graz, Austria

**Keywords:** anaemia, guidelines, Patient Blood Management, preoperative medicine, red blood cell transfusion

## Abstract

**Background:**

Patient Blood Management (PBM) is a multidisciplinary strategy to optimise patient outcomes by preserving autologous blood and reducing unnecessary transfusion. Although PBM initiatives exist worldwide, the consistency of national guideline recommendations remains unclear. This review compared international PBM programmes and explored reasons for variation.

**Methods:**

A systematic review was conducted in accordance with PRISMA guidelines. In March 2024, Medline, Cochrane Library, and Google Scholar were searched for publications from the past 20 years, supplemented by governmental and institutional sources. Guidelines were assessed using the International Centre for Allied Health Evidence checklist. Laws, nationally implemented guidelines, or documents with ministerial involvement were included for a comparative text analysis if they addressed defined PBM elements, were freely accessible, and met quality criteria. The review was registered on PROSPERO and completed in 2025.

**Results:**

Forty national and institutional guidelines were analysed. The greatest variability concerned strategies to reduce diagnostic blood loss. In contrast, most other PBM domains showed substantial agreement, particularly regarding restrictive transfusion strategies, most defined by a haemoglobin threshold of 7 g dl^−1^ (22 guidelines) or lower (nine guidelines). National standardisation was frequently absent, even in countries with advanced hospital-level PBM programmes. Guidance for acute care and intensive care was usually issued by specialty societies rather than national authorities; prehospital recommendations were scarce.

**Conclusions:**

National guidelines on Patient Blood Management remain fragmented, as comprehensive policy frameworks and multidisciplinary coordination are lacking. Greater harmonisation and structured change management are required to support wider global adoption of PBM.

**Systematic review protocol:**

CRD420251032664 (PROSPERO).

**Editor’s key points**

• Patient Blood Management (PBM) is a multidisciplinary strategy to optimise patient outcomes by preserving autologous blood and reducing unnecessary transfusion, but the consistency of national guideline recommendations remains unclear.
• This systematic review compared international PBM programmes and explored reasons for variation.
• Of the 40 national and institutional guidelines analysed, other than strategies to reduce diagnostic blood loss, most PBM domains showed substantial agreement, particularly regarding restrictive transfusion strategies.
• Guidelines remain fragmented, without comprehensive policy frameworks and multidisciplinary coordination, which are required for wider global adoption.

According to the Global Definition Group, Patient Blood Management (PBM) is a patient-centred, systematic, evidence-based approach to improve patient outcomes by managing and preserving a patient’s own blood while promoting patient safety and empowerment.^1^ It is based on three pillars: 1) optimising red cell mass, 2) minimising blood loss, and 3) harnessing and optimising physiological tolerance of anaemia.^2^ These strategies aim to reduce unnecessary blood or blood cell transfusions and costs while maintaining, or ideally improving, clinical outcomes. Against the backdrop of global demographic shifts, increasing demand for blood products, and growing concerns regarding the safety and sustainability of blood supplies, the widespread adoption of PBM should become a global health priority.^3^^,^^4^ The World Health Organisation (WHO) and the European Commission, among other international organisations, have repeatedly called for national-level implementation of PBM strategies.^4^, ^5^, ^6^, ^7^, ^8^ Nevertheless, by 2025, the global uptake of PBM remains inconsistent, with only a limited number of countries having national guidelines. 9, 10, 11, 12, 13, 14, 15, 16, 17, 18, 19, 20, 21, 22, 23, 24, 25, 26, 27, 28, 29, 30, 31, 32, 33, 34, 35, 36, 37, 38, 39, 40, 41, 42, 43, 44, 45, 46, 47, 48, 49, 50, 51, 52, 53, 54 According to the WHO global status report 2022, 73% of member states reported having a national blood policy, but only 59% had a national strategic plan for blood safety, 66% had specific legislation regulating transfusion safety and product quality, and 58% had national advisory committees supporting ministries of health.^55^ Anaemia, often a manifestation of chronic underlying disease, remains highly prevalent, with an estimated 1.95–2.36 billion individuals affected worldwide in 2021, particularly in low- and middle-income countries (LMICs).^7^

Red blood cell (RBC) transfusions are frequently administered in the perioperative setting, especially among patients with preoperative anaemia. Our review aimed to assess the global status of national PBM frameworks, compare existing recommendations, and evaluate their alignment with WHO principles and international best practices. The review also examined whether and how PBM principles have been incorporated into intensive or acute care and prehospital settings. You can find a panel discussing our research in context in Supplementary File S1.

## Methods

### Search strategy and selection criteria

**Fig 1:**
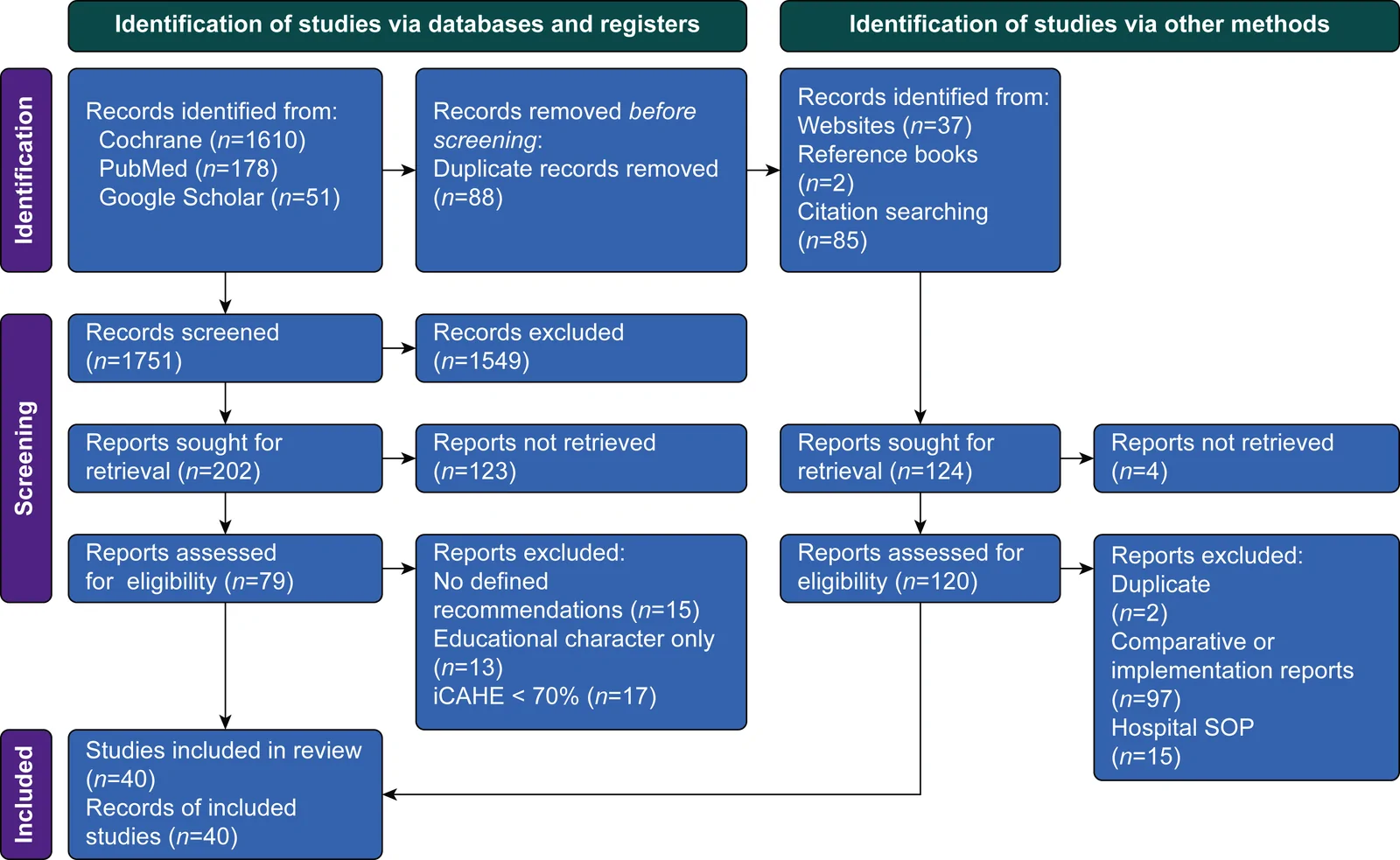
Study selection - PRISMA 2020 flow diagram. Source: Page MJ, *et al.* BMJ 2021;372:n71. https://doi.org/10.1136/bmj.n71. This work is licensed under CC BY 4.0. To view a copy of this licence, visit https://creativecommons.org/licenses/by/4.0/

A systematic review was conducted in accordance with the PRISMA 2020 statement, and the protocol was registered with PROSPERO (CRD420251032664). We searched PubMed, Cochrane Library, and Google Scholar in March 2024; complete search terms are listed in Supplementary File S2. The review focused primarily on directives and legal frameworks related to PBMs and secondarily on PBM or PBM-related guidelines published over the past 20 years. Eligible documents were issued by national health authorities, ministries, professional societies, or national bodies or if their title or abstract suggested an effort to establish national PBM recommendations. Titles and abstracts were screened independently by two authors (DD and GH). For records that identified countries with reported PBM development, a targeted extended search was conducted using citation tracking and handsearching (Fig. 1).

To be eligible for inclusion and quality assessment, documents had to fulfil the following criteria:

• be enshrined in national legislation
• if not legislated, be developed with governmental involvement, as indicated in the document introduction
• and/or be based on a systematic review of evidence, developed by a dedicated clinical working group in anaesthesiology, critical care medicine, transfusion medicine, or haematology
• have national or territorial applicability, as indicated in the title or abstract
• represent the most recent version published.

Documents that fulfilled these criteria were subsequently screened for their coverage of a predefined set of PBM elements, categorised according to the three WHO-defined pillars:

• Pillar 1: perioperative anaemia diagnostics; treatment with iron, erythropoietin (EPO), erythropoietin-stimulating agents (ESAs), and A-B-C-K-vitamins; postponement of elective surgery.
• Pillar 2: minimising diagnostic blood loss; bleeding risk definition of interventions; acute normovolaemic haemodilution (ANH); mechanical autotransfusion (MAT); point-of-care testing (POCT); massive haemorrhage/massive transfusion protocol; management of perioperative antithrombotic therapy or coagulopathy.
• Pillar 3: defined RBC-transfusion thresholds; single-unit transfusion policy; transfusion indications for plasma/cryoprecipitate/factor concentrates/platelets/white blood cells; transfusion indicators in addition to haemoglobin (Hb)/haematocrit (Hct) values.

As a final step in the inclusion process, the practice guidelines’ quality was assessed using the Guideline Quality Checklist published by the International Centre for Allied Health Evidence (iCAHE).^56^^,^^57^ This tool comprises 14 binary items assessing the guideline’s availability, dates, underlying evidence, developers, purpose and intended users, and ease of use. It has been shown to offer comparable accuracy to other quality assessment instruments while being pragmatic and easy to apply. In the absence of further data, establishing quality thresholds to categorise guidelines as high, moderate, or low quality remains challenging. Existing literature provides examples of approaches in which guidelines scoring above 70% are considered high quality.^58^ Legal texts were considered in their original form without formal quality assessment, as evaluation against legal standards was outside the scope of this study.

Documents were excluded if they:

• were developed without governmental involvement or by working groups outside anaesthesiology, critical care medicine, transfusion medicine, or haematology (e.g. gynaecologic, paediatric societies)
• did not contain recommendations addressing any of the predefined PBM elements
• could not be obtained free of charge via university access (e.g. studies from Chest, AABB book series)
• were implementation or monitoring guidelines
• and were purely descriptive, without containing systematically developed statements
• had an iCAHE quality score <10/14 (=71%).

National guidelines available only in the respective national language were translated into English using DeepL or Google Translate (as of July 2024). These were included in the informative section and, when relevant to the predefined content domains, in the final comparative analysis.

Disagreements regarding inclusion were planned to be adjudicated by a third reviewer; however, no disagreements arose that required adjudication.

### Data synthesis and analysis

Following a random external quality check of ∼15% of reports, data for comparative analysis, namely the year of publication, guideline type and recommendations, were manually extracted into a structured spreadsheet and stratified by country and organisation.

Recommendations were subsequently colour-coded according to one of seven predefined categories: pre-interventional patient optimisation; perioperative haemostasis and anaemia management; evaluation of coagulation status; general coagulation management; transfusion practices; education and training; and organisational aspects.

**Fig 2:**
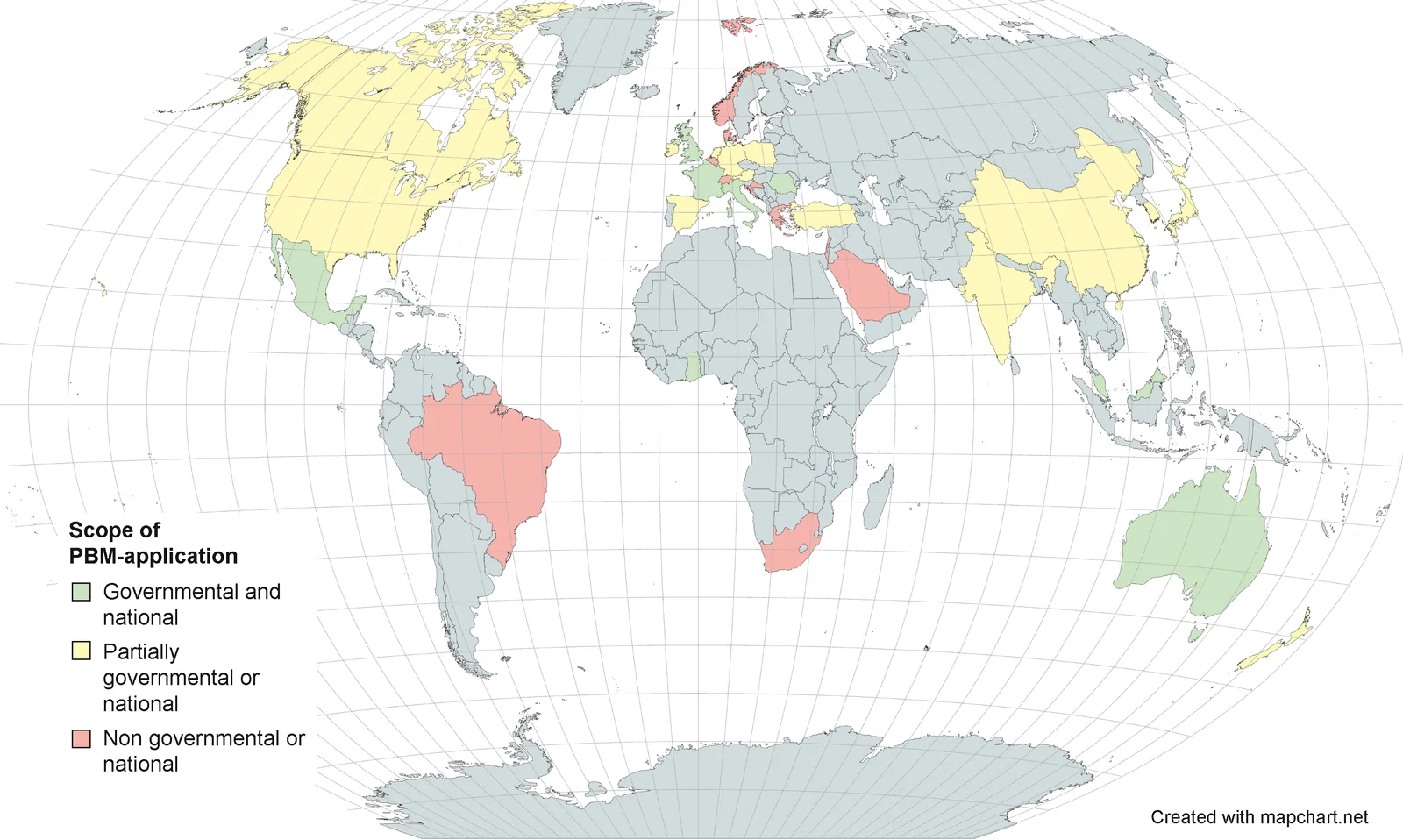
Geographical distribution of included studies and their scope of application.

Final comparisons were performed using a results table generated by systematic tabulation of the selected PBM elements from the working dataset. Where discrepancies were identified, the full texts of the respective guidelines were reviewed in detail to perform a comparative analysis. Alpha-3 country codes were assigned according to ISO 3166-1:2020.^59^ Geographical distribution (Fig. 2) was visualised using MapChart. Because most guidelines included an internal risk-of-bias assessment, no additional appraisal was undertaken. Findings were categorised according to the three PBM pillars and synthesised narratively.

## Results

A total of 1963 records were initially identified. After removal of duplicates and exclusion of documents that were nonspecific to the topic, educational in nature, hospital- or economy-focused reports, or guidelines with an iCAHE quality score below 10/14 (=71%), 40 documents were included for qualitative analysis. Of these, 22 were developed under the leadership or with the involvement of national ministries of health, and some have been formally integrated into national legislation. The remaining documents consist of evidence-based guidelines issued by national or multinational professional societies or constitute clinical practice guidelines. The study’s selection process is summarised in Fig. 1. The calculated quality scores are presented in Supplementary Files S3 and S4.

Australia plays a leading role in the field of PBM, having developed six national guideline modules that are widely referenced internationally.^9^, ^10^, ^11^, ^12^, ^13^, ^14^ Within the broader Asia-Pacific region, Malaysia maintains a national PBM programme, whereas the Democratic People's Republic of Korea, Japan, People's Republic of China and Singapore have integrated specific PBM elements into their national transfusion guidelines.^18^, ^19^, ^20^, ^21^^,^^60^ Several countries, including India, Israel, Lebanon, New Zealand, the People's Republic of China, Saudi Arabia, Singapore and Turkey, are currently developing or adapting PBM-specific protocols.

In Europe, national programmes exist in France, Great Britain, Italy and Romania, and, to a more limited extent in Austria, Poland and the Netherlands.^23^^,^^26^^,^^36^^,^^38^^,^^40^^,^^45^, ^46^, ^47^^,^^61^, ^62^, ^63^ Italy was the first European country with official ministerial PBM support.^64^ In addition, Germany, Great Britain, Ireland, Poland, and the Netherlands have incorporated PBM components into national transfusion guidelines.^24^^,^^35^^,^^36^^,^^47^^,^^62^^,^^65^ Several countries, including Belgium, Denmark, Greece, Ireland, Norway, Spain, and Switzerland, are in the process of developing or revising PBM protocols. No standard national PBM programmes were identified in Croatia or Malta. Besides the European Commission, the WHO and multiple European organisations and societies have released consensus statements, resolutions, educational modules, and transfusion guidelines that promote PBM principles.^5^, ^6^, ^7^, ^8^^,^^28^, ^29^, ^30^, ^31^, ^32^^,^^34^^,^^64^^,^^66^, ^67^, ^68^, ^69^, ^70^, ^71^, ^72^, ^73^, ^74^, ^75^, ^76^, ^77^, ^78^

In Africa, national PBM-related programmes were identified only in Ghana, whereas South Africa is actively developing national protocols.^79^ Both countries have incorporated elements into their national transfusion guidelines.^49^^,^^51^ In addition, South African anaesthesiology and critical care societies have issued specific clinical PBM guidelines.^80^^,^^81^

Across America, Mexico has established a national PBM programme, whereas protocols are being developed in Canada and the USA.^52^^,^^53^^,^^82^^,^^83^ US-American and international societies have published specific guidelines or standards supporting PBM implementation.^84^, ^85^, ^86^, ^87^, ^88^, ^89^, ^90^, ^91^, ^92^, ^93^, ^94^, ^95^, ^96^

From an economic policy perspective, national PBM recommendations are currently present in several high-income (defined by the World Bank definition)^97^ countries (Australia, Austria, France, Germany, Great Britain, Italy, Poland, Romania, and Singapore) and in a smaller number of LMICs (the Democratic People’s Republic of Korea, Malaysia, Mexico, and the People's Republic of China). PBM programmes are under development in additional high-income countries (Belgium, Canada, Denmark, Greece, Ireland, Israel, Japan, New Zealand, Norway, Saudi Arabia, Spain, Switzerland, the Netherlands, and the USA) and in several LMICs (Brazil, Ghana, India, South Africa, and Turkey).

A heatmap of the geographical distribution of included documents and their scope of application is presented in Fig. 2. A detailed scale of country-site and organisational recommendations for the prehospital, perioperative and acute care setting can be found in Supplementary File S3.

A notable variation was observed in guideline scope, content, and target patient populations across countries and organisations (Table 1).^6^, ^7^, ^8^, ^9^, ^10^, ^11^, ^12^, ^13^, ^14^^,^^18^^,^^21^, ^22^, ^23^, ^24^, ^25^, ^26^, ^27^^,^^34^, ^35^, ^36^^,^^40^^,^^42^, ^43^, ^44^, ^45^, ^46^, ^47^^,^^52^^,^^53^^,^^62^, ^63^, ^64^^,^^66^^,^^69^, ^70^, ^71^^,^^73^^,^^74^^,^^76^, ^77^, ^78^^,^^80^^,^^81^^,^^84^, ^85^, ^86^^,^^93^^,^^95^^,^^96^^,^^98^, ^99^, ^100^, ^101^ Most guidelines focus primarily on adult elective surgical patients, whereas recommendations for internal medicine, paediatric, obstetric, acute care, or intensive care populations are inconsistently represented. Guidance for in-hospital acute care is generally issued by anaesthesiology or intensive care societies. Only one pan-European trauma guideline includes elements relevant to the prehospital setting.^69^

**Table 1:**
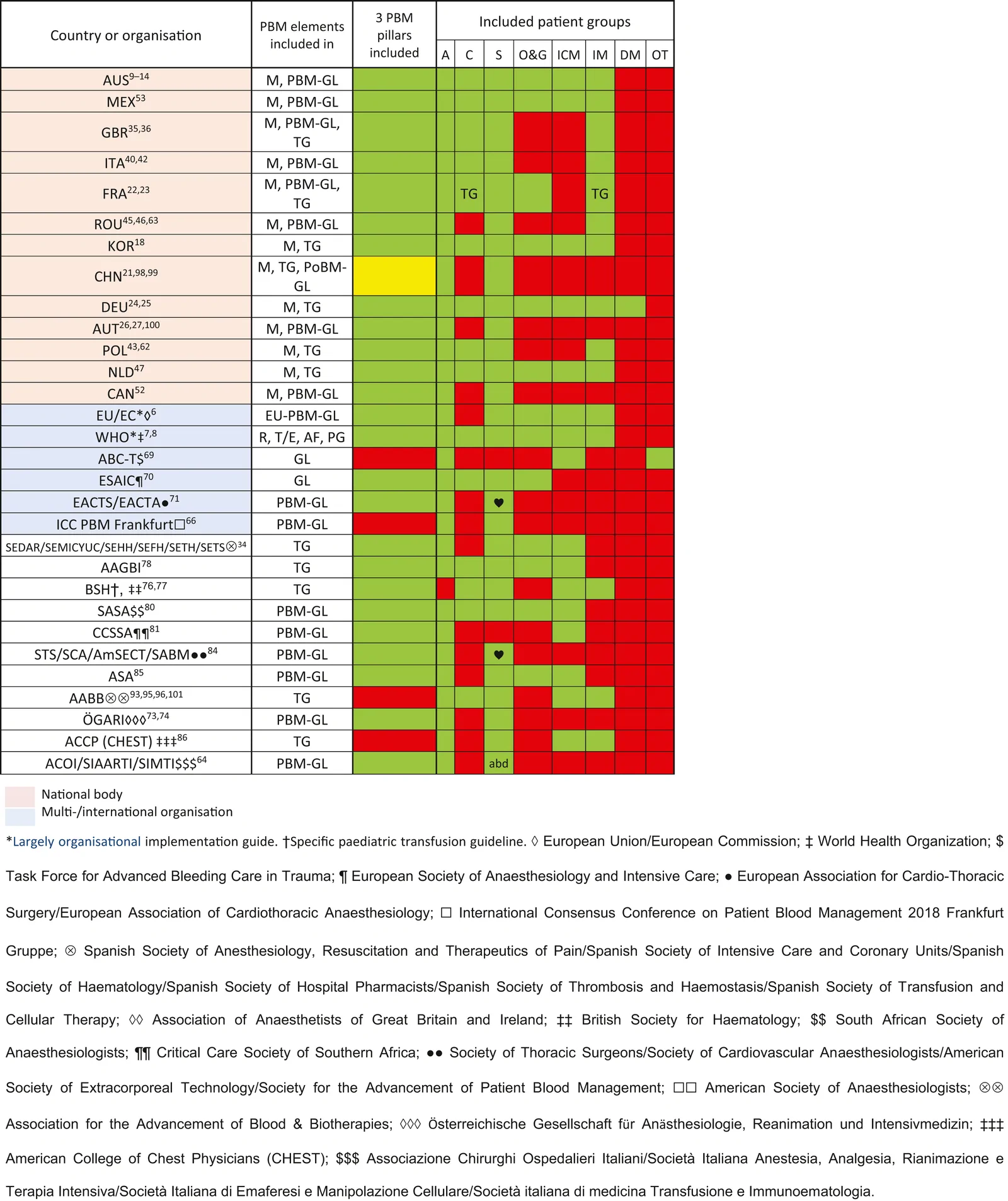
Guidelines, content and patient groups.

Green/yellow/red represent the span from fully to partially to not included. ♥ cardiac surgery. A, Adults; abd, abdominal; AF, Action framework; C, Children; DM, Dental medicine; ICM, Intensive care medicine; IM, Internal medicine (heterogeneous; ranging from one to multiple disciplines, like oncology, haematology, cardiology, nephrology); LL, Guideline; M, Ministerial involvement; O&G, Obstetrics and gynaecology; OT, Ortho-/traumatology; PBM-GL, PBM guideline; PG, Practical guidance; PoBM-LL, Perioperative blood management guideline; R, Resolution; S, Surgery; T/E, Textbook/Education module; TL, Transfusion guideline.^9^, ^10^, ^11^, ^12^, ^13^, ^14^^,^^53^^,^^35^^,^^36^^,^^40^^,^^42^^,^^22^^,^^23^^,^^45^^,^^46^^,^^63^^,^^18^^,^^21^^,^^98^^,^^99^^,^^24^^,^^25^^,^^26^^,^^27^^,^^100^^,^^43^^,^^62^^,^^47^^,^^52^^,^^6^^,^^7^^,^^8^^,^^69^^,^^70^^,^^71^^,^^66^^,^^34^^,^^78^^,^^76^^,^^77^^,^^80^^,^^81^^,^^84^^,^^85^^,^^93^^,^^95^^,^^96^^,^^101^^,^^73^^,^^74^^,^^86^^,^^64^.

In relation to the first PBM pillar (optimising red cell mass), the majority of evaluated guidelines provide comprehensive recommendations on perioperative anaemia diagnostics and treatment, except for the ABC-T and the transfusion guidelines issued by AABB and ACCP (CHEST).^5^^,^^6^^,^^8^^,^^9^^,^^13^^,^^14^^,^^18^^,^^21^^,^^23^^,^^24^^,^^26^^,^^36^^,^^38^^,^^40^^,^^45^, ^46^, ^47^^,^^52^^,^^53^^,^^62^, ^63^, ^64^^,^^66^^,^^69^, ^70^, ^71^^,^^73^^,^^76^, ^77^, ^78^^,^^80^, ^81^, ^82^, ^83^, ^84^, ^85^^,^^102^ Although access to the Chinese national guideline on clinical blood use is restricted, regional publications suggest inclusion of first pillar PBM elements in Chinese guidance.^60^^,^^98^ Several organisations and countries, including ACOI/SIAARTI/SIMTI, SASA, the European Commission, and Italy, explicitly recommend the establishment of anaemia clinics to optimise patients before elective surgery. Most guidelines emphasise the role of thorough history-taking, clinical examinations, and adequate lead time. However, specific protocols for postponing elective procedures until anaemia is corrected vary widely and are often lacking.

For anaemia treatment in iron deficiency, most documents recommend oral iron as first-line therapy. Intravenous iron is reserved for patients with contraindications to oral therapy; intolerance; malabsorption; noncompliance; functional iron deficiency; lack of efficacy; or a clinical need for rapid iron repletion, including those with renal or cardiac comorbidities, patients receiving ESA therapy, or those in the postoperative period.^9^^,^^10^^,^^13^^,^^14^^,^^23^^,^^26^^,^^34^, ^35^, ^36^^,^^40^^,^^42^^,^^47^^,^^52^^,^^62^^,^^74^^,^^81^^,^^84^^,^^100^ A minority of documents recommend intravenous iron as first-line therapy,^22^^,^^53^^,^^64^^,^^70^ whereas in pregnancy and the postpartum period, intravenous iron is frequently advised.^9^^,^^14^^,^^34^^,^^53^^,^^70^ Recommendations regarding EPO, ESAs, and vitamin supplementation show greater variability. Targeted therapy should follow clarification of the underlying cause of anaemia, though the level of procedural detail and the availability of decision-making algorithms differ between guidelines. In South Africa, the Critical Care Society of Southern Africa (CCSSA) critically evaluates the routine use of EPO or iron to reduce transfusion in critically ill anaemic patients, given the lack of a clear mortality benefit and the country’s burden of infection (Table 2).

**Table 2:**
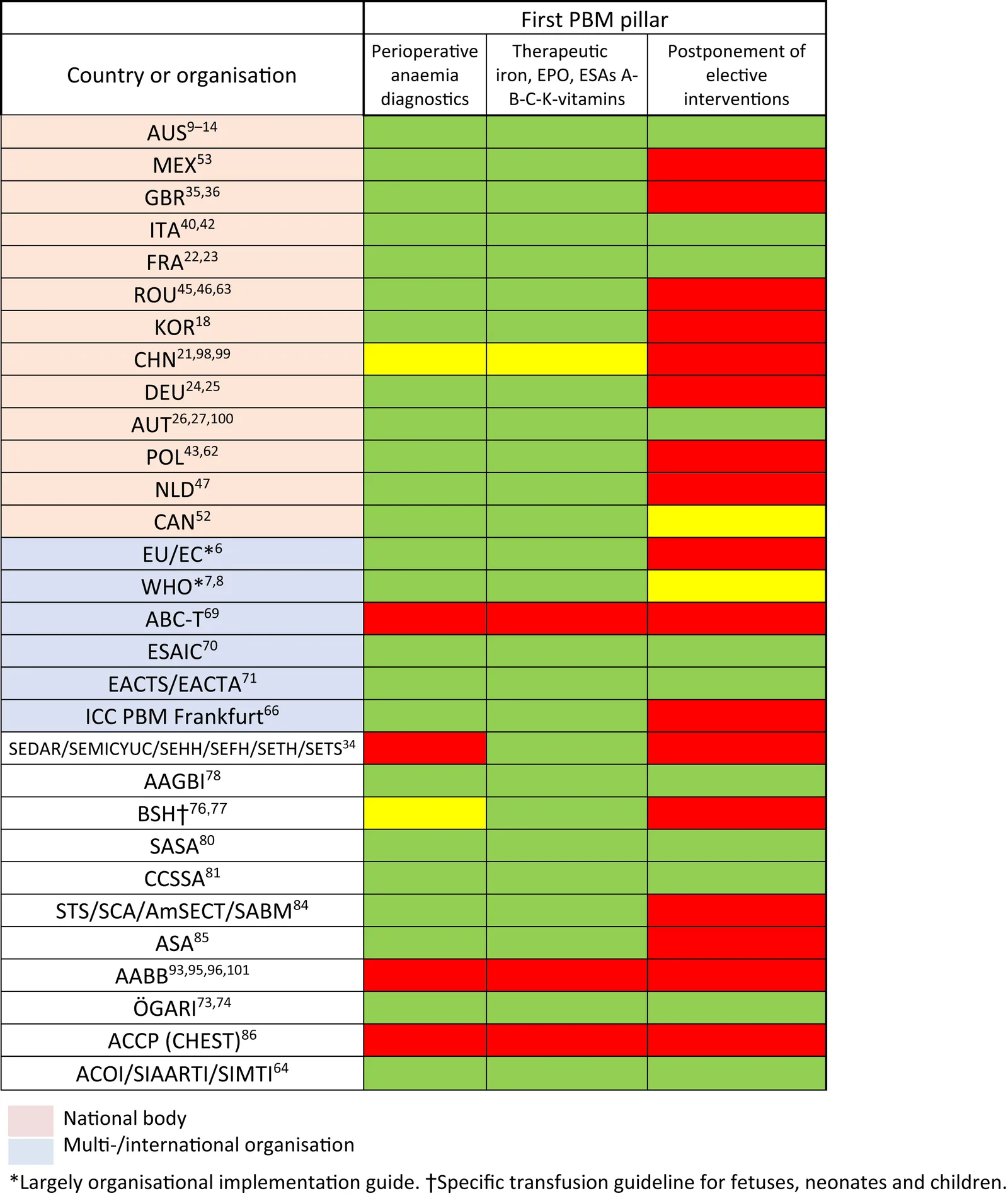
Evaluated subjects of interest - the first pillar of Patient Blood Management.

Green/yellow/red represent the span from fully to partially to not included. Further information regarding the yellow boxes is provided in Supplementary File S5. EPO, erythropoietin; ESA, erythropoietic stimulating agent.^9^, ^10^, ^11^, ^12^, ^13^, ^14^^,^^53^^,^^35^^,^^36^^,^^40^^,^^42^^,^^22^^,^^23^^,^^45^^,^^46^^,^^63^^,^^18^^,^^21^^,^^98^^,^^99^^,^^24^^,^^25^^,^^26^^,^^27^^,^^100^^,^^43^^,^^62^^,^^47^^,^^52^^,^^6^^,^^7^^,^^8^^,^^69^^,^^70^^,^^71^^,^^66^^,^^34^^,^^78^^,^^76^^,^^77^^,^^80^^,^^81^^,^^84^^,^^85^^,^^93^^,^^95^^,^^96^^,^^101^^,^^73^^,^^74^^,^^86^^,^^64^.

Regarding the second PBM pillar (minimising blood loss and bleeding), which encompasses numerous interdisciplinary interventions, the reviewed guidelines show marked heterogeneity. Substantial variability was observed in recommendations concerning the reduction of diagnostic blood loss (targeted sampling, small-volume collection, noninvasive monitoring) and the definition of surgery-related bleeding risk. Some authors consider the impact of diagnostic blood loss on outcomes in adult patients outside intensive care to be negligible, whereas guidelines encompassing paediatric and ICU patients vary, with several providing no explicit recommendations.^18^^,^^34^^,^^47^^,^^53^^,^^62^^,^^73^^,^^80^^,^^85^^,^^86^^,^^93^^,^^101^

Only a few guidelines address blood-conserving surgical techniques, such as minimally invasive extracorporeal circuits, off-pump coronary artery bypass grafting, video-assisted procedures, the use of electrocautery, ultrasound dissectors, argon plasma coagulation, embolisation and tourniquets, as well as avoidance of routine suction drainage.^26^^,^^36^^,^^38^^,^^40^^,^^47^^,^^61^^,^^64^^,^^71^^,^^73^ In contrast, guidance on the use of POCT, including viscoelastic haemostatic assays, is consistently reported across documents, as well as recommendations regarding autologous transfusion techniques, including perioperative autologous donation (PAD), acute normovolaemic haemodilution (ANH), and perioperative cell salvage or machine autotransfusion (MAT). Although routine PAD is not recommended,^9^^,^^45^ some guidelines specify selected indications, such as cardiac surgery, rare blood group phenotypes, complex alloimmunisation, or anticipated difficulty in cross-matching.^18^^,^^24^^,^^26^^,^^38^^,^^40^^,^^42^^,^^47^^,^^53^^,^^62^^,^^73^

Most documents include clinical and organisational frameworks for massive haemorrhage protocols, often detailing activation criteria and treatment steps. In addition, several guidelines recommend intraoperative permissive hypotension or the cautious use of vasopressors to reduce bleeding and transfusion needs.^21^^,^^26^^,^^69^^,^^73^^,^^78^^,^^80^ Management of perioperative antithrombotic therapy and coagulopathy has gained prominence, reflecting the evolving demographic profile of patients. Mechanical thromboprophylaxis as a bridge to medication and physiotherapy is mentioned in a few publications.^65^^,^^69^ (Table 3).

**Table 3:**
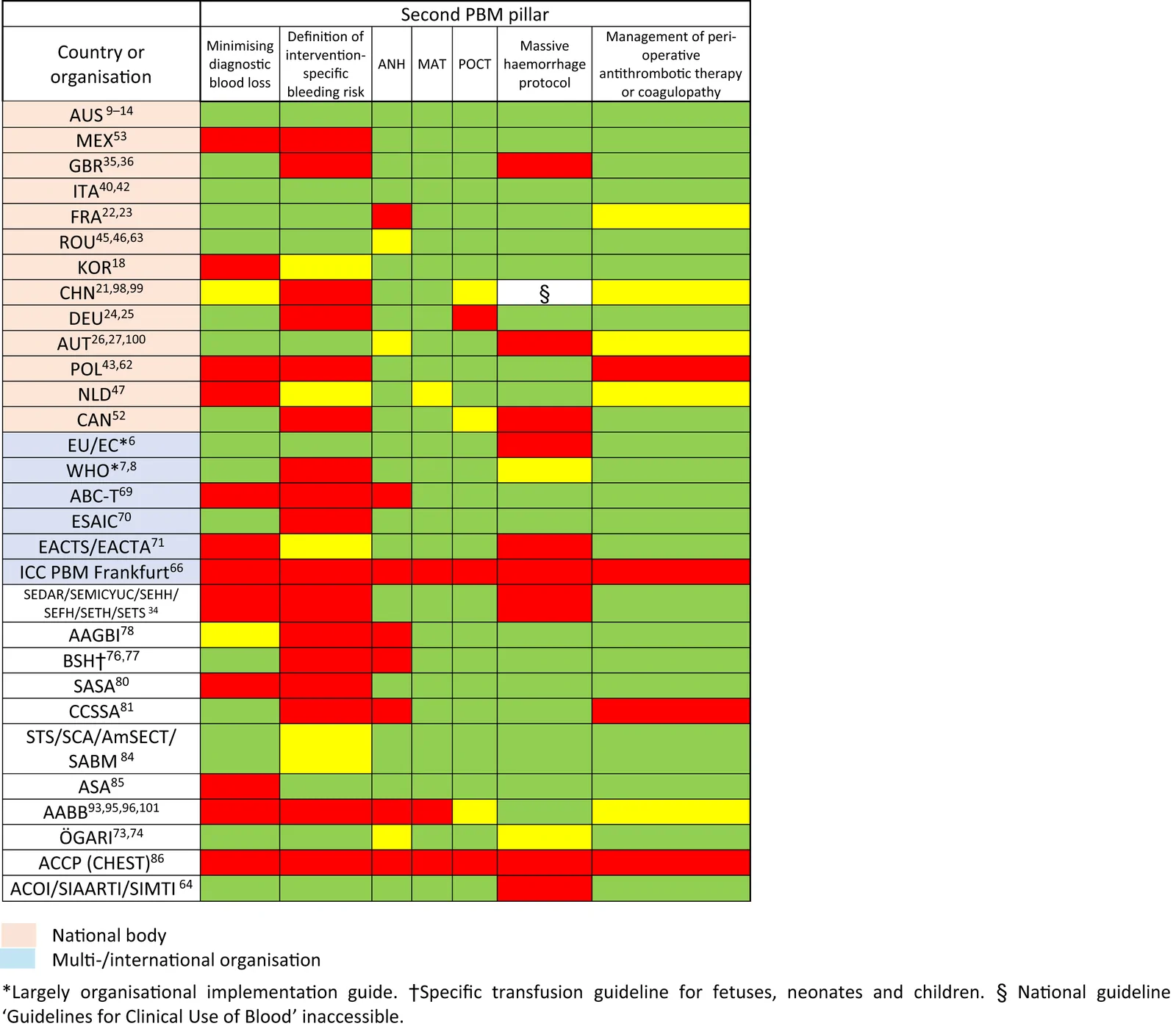
Evaluated subjects of interest - the second pillar of Patient Blood Management.

Green/yellow/red represent the span from fully to partially to not included. Further information regarding the yellow boxes is provided in Supplementary File S5. ANH, acute normovolemic haemodilution; MAT, machine autotransfusion; POCT, point-of-care testing^9^, ^10^, ^11^, ^12^, ^13^, ^14^^,^^53^^,^^35^^,^^36^^,^^40^^,^^42^^,^^22^^,^^23^^,^^45^^,^^46^^,^^63^^,^^18^^,^^21^^,^^98^^,^^99^^,^^24^^,^^25^^,^^26^^,^^27^^,^^100^^,^^43^^,^^62^^,^^47^^,^^52^^,^^6^^,^^7^^,^^8^^,^^69^^,^^70^^,^^71^^,^^66^^,^^34^^,^^78^^,^^76^^,^^77^^,^^80^^,^^81^^,^^84^^,^^85^^,^^93^^,^^95^^,^^96^^,^^101^^,^^73^^,^^74^^,^^86^^,^^64^.

Most guidelines show high concordance regarding the third PBM pillar (harnessing and optimising physiological reserve of anaemia). There is broad agreement that transfusion decisions should not be based solely on Hb or Hct values but should instead reflect a comprehensive clinical assessment. This includes physiological reserve, expected disease trajectory, and medical history. Restrictive transfusion thresholds are consistently recommended. For adult patients, an Hb threshold of 7 g dl^−1^ or lower is frequently cited. Several guidelines, including those from Mexico, Romania, the Democratic People’s Republic of Korea, Germany, Austria, Poland, ESAIC, ASA, and ÖGARI, emphasise that even at these thresholds, transfusion is not mandatory if adequate compensatory mechanisms are present.^18^^,^^24^^,^^26^^,^^45^^,^^46^^,^^53^^,^^62^^,^^63^^,^^70^^,^^73^^,^^85^ For neonates and infants, higher threshold values are suggested depending on clinical condition and the need for organ support.^24^^,^^47^^,^^62^^,^^76^^,^^77^ If a general upper transfusion limit is specified for adults, this is usually set at 10 g dl^−1^ Hb, although deviations are possible in specific cases. Between these lower and upper thresholds, most guidelines define specific targets according to patient groups, pathologies, clinical course, or level of medical support. In acute brain injury and acute coronary syndrome (ACS), thresholds are generally set slightly higher, even within a restrictive transfusion strategy. For instance, the National Blood Authority (NBA) recommends 8 g dl^−1^ for ACS, the European Society of Cardiology suggests 7 g dl^−1^ for stable patients, and the American College of Physicians advises 7–8 g dl^−1^ for hospitalised patients with coronary heart disease.^103^ Some guidelines, such as those from Austria and ÖGARI, define distinct transfusion thresholds before and after surgical haemostasis, whereas ESAIC differentiates between pre- and postoperative anaemic Hb-threshold levels.

Recommendations for a single-unit transfusion policy vary across institutions. This variation is reflected in the ongoing efforts of the national Choosing Wisely initiatives (‘Why give two when one will do?’) and does not apply to paediatric recommendations, in which weight-adjusted volumes are used. Beyond RBC transfusion, the PBM-containing transfusion guidelines also specify indications for blood and blood components, such as plasma, cryoprecipitate, factor concentrates, platelets, white blood cells, or others. These products are also primarily recommended for restrictive use (Table 4).

**Table 4:**
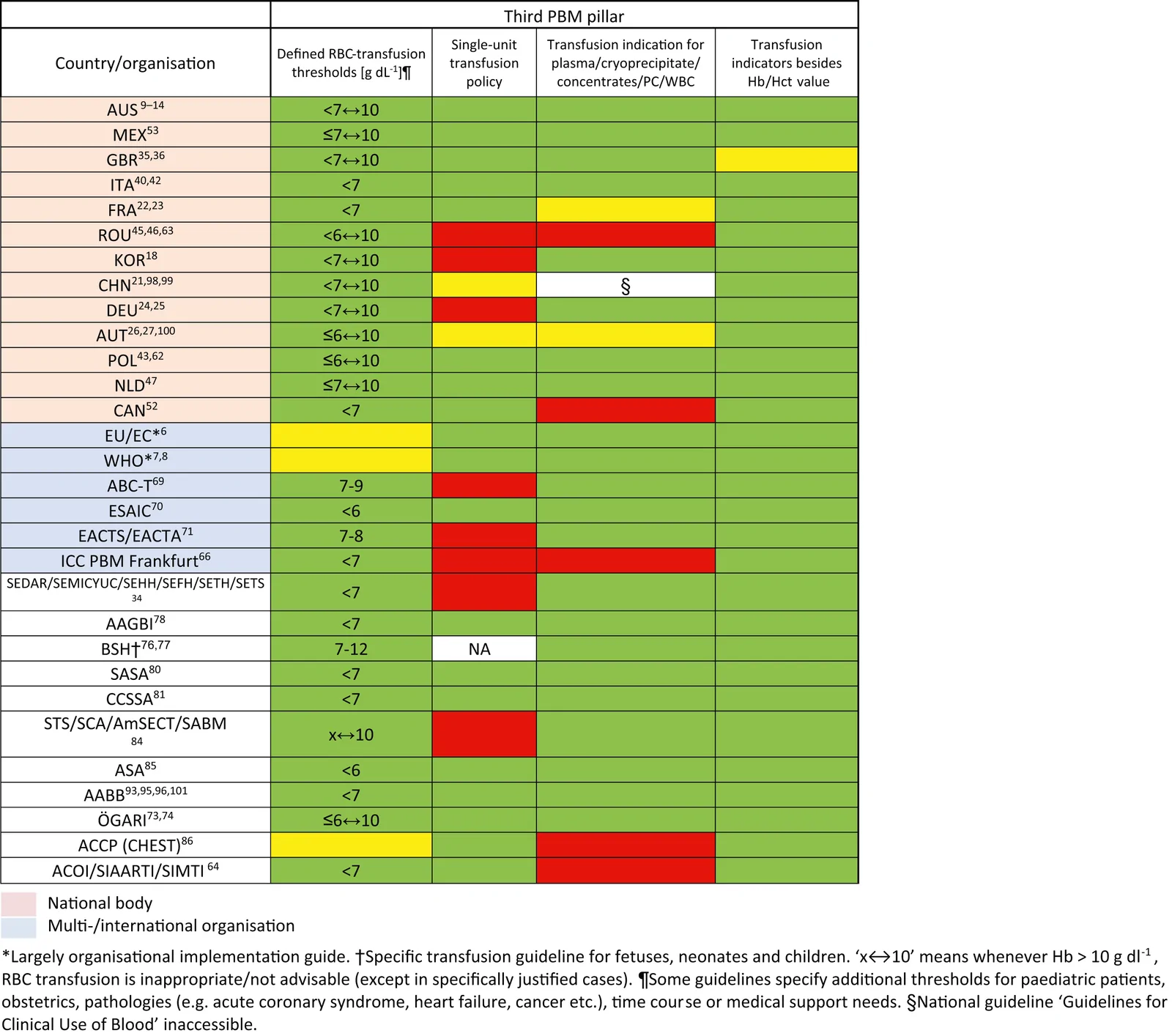
Evaluated subjects of interest - the third pillar of Patient Blood Management.

Green/yellow/red represent the span from fully to partially to not included. Further information regarding the yellow boxes is provided in Supplementary File S5. Hb, heamoglobin, Hct, haematocrit; NA, not applicable; PC, platelet concentrate; RBC, red blood cell; WBC, white blood cell.^9^, ^10^, ^11^, ^12^, ^13^, ^14^^,^^53^^,^^35^^,^^36^^,^^40^^,^^42^^,^^22^^,^^23^^,^^45^^,^^46^^,^^63^^,^^18^^,^^21^^,^^98^^,^^99^^,^^24^^,^^25^^,^^26^^,^^27^^,^^100^^,^^43^^,^^62^^,^^47^^,^^52^^,^^6^^,^^7^^,^^8^^,^^69^^,^^70^^,^^71^^,^^66^^,^^34^^,^^78^^,^^76^^,^^77^^,^^80^^,^^81^^,^^84^^,^^85^^,^^93^^,^^95^^,^^96^^,^^101^^,^^73^^,^^74^^,^^86^^,^^64^.

## Discussion

Our review identified a substantial gap in the availability of formally established, independent national PBM programmes encompassing all three pillars across the pre-, intra-, and postoperative phases and providing interprofessional recommendations, even in HICs. Using our methodology, 13 nations (including nine HICs) were identified as having established such programmes to varying degrees, whereas 14 high-income and 5 LMICs are currently in the process of developing such programmes. These findings are consistent with previously published evidence.^104^, ^105^, ^106^, ^107^, ^108^, ^109^ Only one guideline refers to PBM in the prehospital setting, highlighting a major knowledge gap. Although PBM concepts and blood conservation strategies were introduced in the early 2000s, international organisations, including WHO and the European Commission, have repeatedly called for broader and more uniform implementation of PBM programmes.^4^, ^5^, ^6^, ^7^ Although guidelines *per se* have limited influence on adherence to recommendations and might not accurately reflect PBM activity, those developed with ministerial involvement (such as in Australia, France, Germany, Italy, Mexico, and Romania) establish a mandatory and uniform standard nationwide. Our study also found that in some countries, including Canada, Spain, South Africa, and the USA, professional societies exert considerable influence at the national level despite the current absence of formal legal authority.

Only a minority of included guidelines addressed all three pillars of PBM. Among those that did, the highest degree of concordance was observed for recommendations related to the third pillar, followed by the first. By contrast, greatest variability occurred in measures linked to the second pillar, particularly in strategies to reduce diagnostic blood loss. Deviations were evident not only between national and international guidelines but also between governmental and societal recommendations, a phenomenon previously reported in other clinical fields.^110^ Definitions of interventional bleeding risk were often y unspecified. National transfusion guidelines were more likely than PBM-specific documents to include recommendations on plasma, cryoprecipitate, factor concentrates, platelets, and white blood cell transfusions within restrictive transfusion frameworks.

We interpret the variation observed across guidelines as reflecting the inherently complex and interdisciplinary nature of PBM. Elements of the second pillar apply across community and hospital settings, in both surgical and nonsurgical care, and in acute as well as peri-interventional contexts, each involving different stakeholder groups. Geopolitical, cultural, and health system factors, together with differences in methodology, evidence appraisal, and consensus processes, are likely to contribute to the heterogeneity of national recommendations. Variation in publication dates and the underlying evidence base further contribute to this heterogeneity, even though some guidelines reference one another.

We propose three approaches to address this issue. Discrepancies between national guidelines and those issued by professional associations could be reduced by keeping national framework guidelines general, with speciality societies providing more specific supplementary recommendations. Alternatively, a national clinical practice guideline could be developed with the active participation of all relevant professional associations; however, this approach risks producing excessively long and less practical documents. Some of the guidelines analysed have already exceed 500 pages. Differences related to publication date might in the future be mitigated by adopting living guideline models.^111^

Our study has several limitations. First, we conducted a systematic literature review rather than directly surveying national ministries or WHO member states, which might have limited completeness. Second, our analysis was limited to predefined elements from the three PBM pillars, introducing potential selection bias. Third, most retrieved documents originated from HICs and were published in English, raising the possibility of language and publication bias. Systematic reviews that restrict inclusion to English-language publications inevitably introduce a language-related bias and limit global representation. Although only about one-fifth of the global population speaks English as a first or second language, nearly three-quarters of scientific publications are published in English. Relevant non-English documents are frequently confined to the grey literature and might be identifiable only through handsearching or searches conducted in the original language, thereby reducing their visibility when using conventional search strategies.^112^ Country income-level bias also remains an important consideration. From a global perspective, facilitating knowledge transfer from HICs to LMICs with high transfusion rates is likely to offer substantial potential benefit. Although national PBM recommendations in LMICs are increasingly aligned through international collaboration, including WHO-led initiatives and adaptation of HIC guidelines, implementation remains constrained by limited resources. Inadequate infrastructure, insufficient funding, and deficits in training and continuing professional education represent key barriers.^107^ Fourth, the multidisciplinary nature of PBM and the methodological heterogeneity of the documents complicated both the search strategy and qualitative comparison. Definitions of PBM varied across initiatives, despite the availability of international templates (e.g. from WHO or the European Commission).^1^ Finally, several included guidelines date back to 2010 and are currently under revision; although recent developments were considered where available, updated versions remain pending.

In conclusion, despite calls for the introduction and promotion of national PBM principles for more than 25 years, only a limited number of national guidelines exist worldwide. Where they do exist, their scope and recommendations vary, most likely reflecting local contexts and underlying evidence. Although differences between national and speciality-specific PBM or transfusion guidelines are relatively minor, they remain relevant and reflect that one approach does not apply to all situations and contexts.

## Authors’ contributions

DD: conceptualisation, data curation, formal analysis, investigation, methodology, project administration, resources, software, validation, visualisation, writing - original draft, writing - review and editing

GH: conceptualisation, data curation, methodology, investigation supervision, validation, writing - original draft, writing - review and editing

DA: conceptualisation, supervision, validation, visualisation, writing - review and editing

LH, MR: conceptualisation, supervision, validation, writing - review and editing

PZ: conceptualisation, supervision, validation, formal analysis, project administration, supervision, validation, writing - review and editing

HBC: conceptualisation, data curation, investigation, methodology, supervision, validation, writing - review and editing

All authors agree to be accountable for all aspects of the work.

## Declaration of Generative AI and AI-assisted technologies in the writing process

During the preparation of this work the authors used ChatGPT to help with grammar and style. After using this tool, the authors reviewed and edited the content as needed and take full responsibility for the content of the publication.

## Declaration of interest

The authors declare that they have no conflict of interest.
