# Supplementary material for: Patient Blood Management: a systematic review of current international guidelines
Source: Br J Anaesth. 2026 May 12;137(1):74–88. doi: 10.1016/j.bja.2026.03.055 (PMC13494083; doi:10.1016/j.bja.2026.03.055)
Supplement: Supplementary file 1 [file mmc1.docx]

*Panel:* **Research in context**

**Evidence before this study**

Previous research has examined Patient Blood Management mainly at the level of single hospitals or individual countries. Despite international frameworks like those from the World Health Organization, PBM-programs appeared highly variable worldwide.

**Added value of this study**

Despite repeated calls from international health authorities, including WHO, the global uptake of PBM remains limited. To compare national approaches systematically, we conducted a systematic review of PubMed, the Cochrane Library, and Google Scholar for articles published between March 2005 and 2024.

National guidelines show modest variation, particularly in recommendations to minimize blood loss. Most guidelines focus on elective surgical settings, whereas recommendations for intensive care or acute care are inconsistently addressed, and guidance for prehospital settings is largely absent.

**Implications of all the available evidence**

Context-specific factors and willingness to change appear central to PBM introduction. A coordinated approach - combining governmental endorsement with professional engagement – seem essential to achieve broader and more effective adoption worldwide.
