# Supplementary material for: Patient Blood Management: a systematic review of current international guidelines
Source: Br J Anaesth. 2026 May 12;137(1):74–88. doi: 10.1016/j.bja.2026.03.055 (PMC13494083; doi:10.1016/j.bja.2026.03.055)
Supplement: Supplementary file 2 [file mmc2.docx]

**Full search terms for Cochrane, PubMed and Google Scholar**

((PBM) or ("patient blood management") or (transfusion)) and ((national) or (guideline) or (minister) or (ministry) or (government) or (clinical practice) or (implementation)) in Title Abstract Keyword - with Publication Year from 2005 to present, with Cochrane Library publication date from Mar 2005 to present, in Trials - Cochrane Central Register of Controlled Trials, Issue 2 of 12, February 2024, 10.03.2024 (Cochrane);

((PBM) or ("patient blood management") or (transfusion)) and ((national) or (guideline) or (minister) or (ministry) or (government) or (clinical practice) or (implementation)) – Filters applied: Free full text, Government Publication, Guideline, Legal Case, Legislation, Practice Guideline, from 2005 - 3000/12/12, 10.03.2024 (PubMed);

((PBM) or ("patient blood management") or (transfusion)) and ((national) or (guideline) or (minister) or (ministry) or (government) or (clinical practice) or (implementation)) from 2005, 10.03.2024 (Google scholar).
