# Supplementary material for: Patient Blood Management: a systematic review of current international guidelines
Source: Br J Anaesth. 2026 May 12;137(1):74–88. doi: 10.1016/j.bja.2026.03.055 (PMC13494083; doi:10.1016/j.bja.2026.03.055)
Supplement: Supplementary file 3 [file mmc3.docx]

|  | |  | Scope of application | | | Recommendations | | | | |
| --- | --- | --- | --- | --- | --- | --- | --- | --- | --- | --- |
|  | | Country | govern- mental | private +  national | private +  locoregional | pre- hospital | pre- operative | intra- operative | post- operative | acute  setting |
|  | | AUS |  |  |  |  |  |  |  |  |
|  | | IND |  |  |  |  |  |  |  |  |
|  | | TUR |  |  |  |  |  |  |  |  |
|  | | NZL |  |  |  |  |  |  |  |  |
|  | | KOR |  |  |  |  |  |  |  |  |
|  | | SGP |  |  |  |  |  |  |  |  |
|  | | MYS |  |  |  |  |  |  |  |  |
|  | | CHN |  |  |  |  |  |  |  |  |
|  | | FRA |  |  |  |  |  |  |  |  |
|  | | DEU |  |  |  |  |  |  |  |  |
|  | | AUT |  |  |  |  |  |  |  |  |
|  | | ESP |  |  |  |  |  |  |  |  |
|  | | GBR |  |  |  |  |  |  |  |  |
|  | | BEL |  |  |  |  |  |  |  |  |
|  | | ITA |  |  |  |  |  |  |  |  |
|  | | POL |  |  |  |  |  |  |  |  |
|  | | ROU |  |  |  |  |  |  |  |  |
|  | | IRL |  |  |  |  |  |  |  |  |
|  | | NLD |  |  |  |  |  |  |  |  |
|  | | CAN |  |  |  |  |  |  |  |  |
|  | | MEX |  |  |  |  |  |  |  |  |
|  | | USA |  |  |  |  |  |  |  |  |
|  | | GHA |  |  |  |  |  |  |  |  |
|  | PBM-elements comprehensively implemented and established | | | | | | | | | |
|  | Implementation/development/adoption of individual PBM-elements | | | | | | | | | |
|  | Field not targeted | | | | | | | | | |

Table S1 Country-site PBM scope of application and recommendations

|  |  | iCAHE | Scope of application | | | | Recommendations | | | | |  |
| --- | --- | --- | --- | --- | --- | --- | --- | --- | --- | --- | --- | --- |
|  | Organisation | Total % score | govern- mental | private +  national | private +  locoregional | clinical  division | pre- hospital | pre- operative | intra- operative | post- operative | acute  setting |  |
| Asiapacific | NBA WAPBM * module 1 | 100 |  |  |  |  |  |  |  |  |  |  |
|  | NBA WAPBM * module 2 | 93 |  |  |  |  |  |  |  |  |  |  |
|  | NBA WAPBM * module 3 | 93 |  |  |  |  |  |  |  |  |  |  |
|  | NBA WAPBM * module 4 | 93 |  |  |  |  |  |  |  |  |  |  |
|  | NBA WAPBM * module 5 | 93 |  |  |  |  |  |  |  |  |  |  |
|  | NBA WAPBM * module 6 | 93 |  |  |  |  |  |  |  |  |  |  |
| Europe | ICC PBM Frankfurt  2018 group‡ | 93 |  |  |  |  |  |  |  |  |  |  |
|  | NATA§ | 86 |  | international |  |  |  |  |  |  |  |  |
|  | SEDAR/SEMICYUC/ SEHH/SEFH/SETH/ SETS¶ | 79 |  |  |  |  |  |  |  |  |  |  |
|  | ACOI/SIAARTI/ SIdEM/SIMTI†† | 93 |  |  |  |  |  |  |  |  |  |  |
|  | EU/EC‡‡ | - | European |  |  |  |  |  |  |  |  |  |
|  | ABC-T§§ | 93 |  | European |  |  |  |  |  |  |  |  |
|  | ESAIC¶¶ | 93 |  | European |  |  |  |  |  |  |  |  |
|  | EACTS/EACTA*** | 93 |  | European |  |  |  |  |  |  |  |  |
|  | AAGBI‡‡‡ | 71 |  |  |  |  |  |  |  |  |  |  |
|  | BSH§§§ | 86 |  |  |  |  |  |  |  |  |  |  |
|  | WHO¶¶¶ | - | international | |  |  |  |  |  |  |  |  |
|  | FMS● | 79 |  |  |  |  |  |  |  |  |  |  |
|  | HAS◊ | 93 |  |  |  |  |  |  |  |  |  |  |
|  | ÖGARI☐ | 79 |  |  |  |  |  |  |  |  |  |  |
| America | CENETEC●● | 93 |  |  |  |  |  |  |  |  |  |  |
|  | AABB◊◊ | 93 |  | international |  |  |  |  |  |  |  |  |
|  | STS/SCA/AmSECT/ SABM☐☐ | 100 |  |  |  |  |  |  |  |  |  |  |
|  | ASA⮾⮾ | 93 |  |  |  |  |  |  |  |  |  |  |
|  | ACCP (CHEST)●●● | 93 |  |  |  |  |  |  |  |  |  |  |
|  | SASA⮾⮾⮾ | 86 |  |  |  |  |  |  |  |  |  |  |
|  | CCSSA**** | 100 |  |  |  |  |  |  |  |  |  |  |
|  | PBM-elements comprehensively implemented and established | | | | | | | | | | |  |
|  | implementation/development/adoption of individual PBM-elements | | | | | | | | | | |  |
|  | field not targeted | | | | | | | | | | |  |
| * National Blood Authority Western Australia Patient Blood Management Group; ‡ International Consensus Conference on Patient Blood Management 2018 Frankfurt Gruppe; $ Network for the Advancement of Patient Blood Management, Haemostasis and Thrombosis; ¶ Spanish Society of Anesthesiology, Resuscitation and Therapeutics of Pain/Spanish Society of Intensive Care and Coronary Units/Spanish Society of Haematology/Spanish Society of Hospital Pharmacists/Spanish Society of Thrombosis and Haemostasis/Spanish Society of Transfusion and Cellular Therapy; †† Associazione Chirurghi Ospedalieri Italiani/Società Italiana Anestesia, Analgesia, Rianimazione e Terapia Intensiva/Società Italiana di Emaferesi e Manipolazione Cellulare/Società italiana di medicina Transfusione e Immunoematologia; ‡‡ European Union/European Commission; §§ Task Force for Advanced Bleeding Care in Trauma; ¶¶ European Society of Anaesthesiology and Intensive Care; *** European Association for Cardio-Thoracic Surgery/European Association of Cardiothoracic Anaesthesiology; ‡‡‡ Association of Anaesthetists of Great Britain and Ireland; §§§ British Society for Haematology; ¶¶¶ World Health Organization; ● Federatie Medisch Specialisten; ◊ Haute Autorité de Santé; ☐ Österreichische Gesellschaft für Anästhesiologie, Reanimation und Intensivmedizin; ●● Centro Nacional de Excelencia Tecnológica en Salud; ◊◊ Association for the Advancement of Blood & Biotherapies; ☐☐ Society of Thoracic Surgeons/Society of Cardiovascular Anesthesiologists/American Society of Extracorporeal Technology/Society for the Advancement of Patient Blood Management; ⮾⮾ American Society of Anesthesiologists; ●●● American College of Chest Physicians (CHEST); ⮾⮾⮾ South African Society of Anaesthesiologists; **** Critical Care Society of Southern Africa | | | | | | | | | | | |  |
|  |  |  |  |  |  |  |  |  |  |  |  |  |
|  |  |  |  |  |  |  |  |  |  |  |  |  |
|  |  |  |  |  |  |  |  |  |  |  |  |  |
|  |  |  |  |  |  |  |  |  |  |  |  |  |
|  |  |  |  |  |  |  |  |  |  |  |  |  |
|  |  |  |  |  |  |  |  |  |  |  |  |  |

Table S2 Organisational PBM scope of application and recommendations
