# Supplementary material for: Patient Blood Management: a systematic review of current international guidelines
Source: Br J Anaesth. 2026 May 12;137(1):74–88. doi: 10.1016/j.bja.2026.03.055 (PMC13494083; doi:10.1016/j.bja.2026.03.055)
Supplement: Supplementary file 4 [file mmc4.docx]

| **Organisation** | **iCAHE Total % score** |
| --- | --- |
| NOIDA (IND)^1^ | 50 |
| HSA (SGP)^2^ | 64 |
| NZBS (NZL)^3^ | 36 |
| BTWG (MLT)^4^ | 50 |
| MDD (MYS)^5^ | 64 |
| TSCVS/TSC/SCTAIC (TUR)^6^ | 64 |
| CSS (BEL)^7^ | 57 |
| ÖGBT (AUT)^8^ | 43 |
| MAPBM protocolo 1 (ESP)^9^ | 43 |
| MAPBM protocolo 4 (ESP)^10^ | 57 |
| MAPBM protocolo 6 (ESP)^11^ | 43 |
| MAPBM protocolo 7 (ESP)^12^ | 50 |
| MAPBM protocolo 8 (ESP)^13^ | 36 |
| MAPBM protocolo 10 (ESP)^14^ | 57 |
| NTAG (IRL)^15^ | 64 |
| NBS (GHA)^16^ | 43 |
| SANBS/WCBS (ZAF)^17^ | 43 |

Table S1 Documents excluded because of a calculated iCAHE-score <70%

**References**

1	National Institute of Biologicals, Ministry of Health & Family Welfare, Government of India. Good Blood Transfusion Practices - Guidance for Rational use of blood. 2022.

2	Kaur H, Hui Ming T, et al. NATIONAL GUIDELINES ON CLINICAL TRANSFUSION. 2023.

3	New Zealand Blood Service. Transfusion medicine handbook 3rd edition. 2023; published online April. https://www.nzblood.co.nz/healthcare-professionals/transfusion-medicine/transfusion-medicine-handbook/1-introduction/1-3-clinical-practice-guidelines (accessed Jan 29, 2026).

4	Blood Transfusion Working Group. National Standards for Blood Transfusion - Reviewed 2018. 2012.

5	Medical Development Division, Ministry of Health Malaysia. Consensus Statement on Patient Blood Management. 2024.

6	Ertugay S, Kudsioğlu T, Şen T, *et al.* Consensus report on patient blood management in cardiac surgery by Turkish Society of Cardiovascular Surgery (TSCVS), Turkish Society of Cardiology (TSC), and Society of Cardio-Vascular-Thoracic Anaesthesia and Intensive Care (SCTAIC). *Turkish Journal of Thoracic and Cardiovascular Surgery* 2019; **27**: 429–50.

7	PUBLICATION DU CONSEIL SUPERIEUR DE LA SANTE N° 8381 Bonnes pratiques de transfusion à l’usage des hôpitaux. Bruxelles: Coseil Supérieur de la Santé, 2010 [www.css-hgr.be](http://www.css-hgr.be). French

8	Burda G, Hauptlorenz^ S, Höcker P, *et al.* ÖGBT-Standards Klinische Hämotherapie - Revision Oktober 2022 8 ÖGBT Standards Clinical Hemotherapy - Revision October 2022]. 2019; published online June. https://oegbt.clubdesk.com/clubdesk/fileservlet?id=1000133&s=djEtXXUzJ3TgiOcjA-uA39QqyqJIlmt4FeqyVAWnnIIE9AQ= (accessed Jan 29, 2026). German

9	Barquero M, Bisbe E, Basora C, Colomina MaJosé, Jericó C. 1. Protocolo para la optimización preoperatoria de la hemoglobina en la cirurgia programada [1. Protocol for preoperative hemoglobin optimization in scheduled surgery]. In: Protocolos MAPBM, online. MAPBM. https://mapbm.org/protocols/en (accessed Dec 23, 2023). Spanish

10	Abad A, Barquero M, Tió M. 4. Patient Blood Management en urgencias [4. Guideline on Patient Blood Management in Emergency Care]. In: Protocolos MAPBM, online. MAPBM. https://mapbm.org/protocols/en (accessed Dec 23, 2023). Spanish

11	Barquero M, Abad A, Basora C, *et al.* 6. Protocolo sobre el uso del ácido tranexámico [6. Guideline on the Use of Tranexamic Acid]. In: Protocolos MAPBM, online. MAPBM. https://mapbm.org/protocols/en (accessed Dec 23, 2023). Spanish

12	Barquero M, Basora C, Bisbe E, Colomina M, Siera P. 7. Manejo de la hemorragia masiva [7. Guideline for the Management of Massive Hemorrhage]. In: Protocolos MABPM, online. MAPBM. https://mapbm.org/protocols/en (accessed Dec 23, 2023). Spanish

13	Bassas E, Sierra P. 8. Manejo perioperatorio de la medicación antitrombótica [8. Perioperative management of antithrombotic medication]. In: Protocolos MAPBM, online. MAPBM. https://mapbm.org/protocols/en (accessed Dec 23, 2023). Spanish

14	Barquero M, Bisbe E, Basora C, Colomina M, Jericó C. 10. Protocolo de ayuda a la decisión transfusional 10. Protocol for Transfusion Decision Support]. In: Protocolos MAPBM, online. MAPBM. https://mapbm.org/protocols/en (accessed Dec 23, 2023). Spanish

15	Department of Health. Unexpected Intraoperative Life Threatening Haemorrhage (NCEC National Clinical Clinical Guideline No. 29). Dublin: The Department of Health, 2022 https://www.gov.ie/en/collection/c9fa9a-national-clinical-guidelines.

16	National Blood Service Ghana. National Guidelines for the Clinical Use of Blood and Blood Products in Ghana. National Blood Service, 2013 www.nbsghana.org.

17	Bellairs G, Rapodile T, Hilton C, *et al.* CLINICAL GUIDELINES FOR THE USE OF BLOOD AND BLOOD PRODUCTS IN SOUTH AFRICA. SANBS|WCBS, 2023 www.wcbs.org.za.
