# Supplementary material for: Patient Blood Management: a systematic review of current international guidelines
Source: Br J Anaesth. 2026 May 12;137(1):74–88. doi: 10.1016/j.bja.2026.03.055 (PMC13494083; doi:10.1016/j.bja.2026.03.055)
Supplement: Supplementary file 5 [file mmc5.docx]

| **"Yellow box" - Differences in the spotlight** | | | |
| --- | --- | --- | --- |
| Country/  organisation | 1. PBM pillar | | |
|  | Perioperative anaemia diagnostics | Therapeutical iron, EPO, ESAs A-B-C-K-vitamins | Postponement of elective interventions |
| CHN | These are included in local protocols but are not defined within regional or national frameworks. | |  |
| CAN |  |  | "Institutions should have guidelines on postponing surgery until anaemia is corrected." |
| WHO |  |  | "Bearing in mind that PBM means that before surgery every reasonable measure should be taken to optimize […] blood volume, to minimize […] blood loss and to harness and optimize […] physiological tolerance of anaemia." |
| BSH | "Foetal blood counts should be rapidly available […] and a blood film should […] be made to confirm the count and […] diagnosis.” |  |  |

Table S1 Differences in the spotlight - 1. PBM pillar

|  | **"Yellow box" - Differences in the spotlight** | | | | | | | | |
| --- | --- | --- | --- | --- | --- | --- | --- | --- | --- |
|  | Country/ organisation | 2. PBM pillar | | | | | | | |
|  |  | Minimising diagnostic blood loss | Definition of intervention-specific bleeding risk | | ANH | MAT | POCT | Massive haemorrhage protocol | Management of peri-operative antithrombotic therapy or coagulopathy |
|  | FRA |  |  |  | |  |  |  | References to other guidelines |
|  | ROU | Referring to: ANH:  Listed in Table I (Elements of a Patient's Blood Management Programme) without further explanation. | | | | | | | |
|  | KOR | Referring to: Definition of intervention-specific bleeding risk:  "[…] patients undergoing high-risk surgeries such as heart surgery and spine surgery." | | | | | | | |
|  | CHN | Referring to: Definition of intervention-specific bleeding risk:  In Hong Kong: "A PBM framework should be developed [...], including [...] optimisation of patients’ haematopoiesis […]."  Referring to: POCT:  "Clinicians [...] should [...] correctly apply mature clinical blood transfusion technology and blood protection technology." In Hong Kong: "[...] and application of [...] point-of-care testing to optimise patients’ outcomes with less transfusion."  Referring to: Management of peri-operative antithrombotic therapy or coagulopathy:  In Hong Kong: "A PBM framework should be developed [...], including [...] correction of coagulation before surgical procedures." | | | | | | | |
|  | AUT | Referring to: ANH:  Mentioned once in the text as alternative to autologous blood donation  Referring to Management of peri-operative antithrombotic therapy or coagulopathy:  "In patients with […] coagulation disorders and […] pathological blood coagulation findings, interdisciplinary optimization should be initiated preoperatively." | | | | | | | |
|  | NLD | Referring to: Definition of intervention-specific bleeding risk:  Individual examples with high risk (e.g. cardiac surgery, correction of congenital craniosynostosis/scoliosis etc.) described  Referring to: MAT  Mentioned once in the text as a technique to reduce blood loss and transfusions.  Referring to: Management of peri-operative antithrombotic therapy or coagulopathy:  Recommendations provided on plasma transfusion for patients with hepatic impairment requiring surgery, with vitamin K deficiency, or discontinuation of oral anticoagulants.  Thrombocytopathy and anticoagulant management are addressed by reference to specific guidelines | | | | | | | |
|  | CAN | Referring to: POCT:  Mentioned once in the text as a component of a PBM program/laboratory resource | | | | | | | |
|  | WHO | Referring to: Massive haemorrhage protocol:  "Although guidelines are available [...] clinical application of these guidelines in direct patient care is underutilized." | | | | | | | |
|  | EACTS/EACTA | Referring to: Definition of intervention-specific bleeding risk:  Recommended that the multidisciplinary team discusses the optimal surgical strategy based on the anticipated bleeding risk, among other factors | | | | | | | |
|  | AAGBI | Referring to: Minimising diagnostic blood loss:  Noted in text that in critical care, blood loss and sampling, among other factors, may be important contributors | | | | | | | |
|  | STS/SCA/AmSECT/SABM | Referring to: Definition of intervention-specific bleeding risk:  The role of bleeding risk attributable to a particular procedure is noted in text. Cardiac surgery is classified as high-risk. | | | | | | | |
|  | AABB | Referring to: POCT:  Recommendation for future research  Referring to: Management of peri-operative antithrombotic therapy or coagulopathy:  Recommendations for plasma and platelet transfusion in patients with coagulopathy | | | | | | | |
|  | ÖGARI | Referring to: ANH:  Mentioned once in the text as alternative to autologous blood donation  Referring to: Massive haemorrhage protocol:  Promotion of haemostasis according to hospital-specific treatment algorithms, including coagulation laboratory guidance, empirical coagulation therapy, and proposals for ROTEM-based algorithms | | | | | | | |

Table S2 Differences in the spotlight - 2. PBM pillar

| "Yellow box" - Differences in the spotlight | | | | |
| --- | --- | --- | --- | --- |
| Country/ organisation | 3. PBM pillar | | | |
|  | defined RBC-transfusion thresholds [g/dL^-1^]¶ | Single-unit  transfusion policy | Transfusion indication for plasma/cryoprecipitate/ concentrates/PC/WBC | Transfusion indicators besides Hb-/Hct-value |
| GBR |  |  |  | Noted clinical assessment alongside guideline-based triggers and identified a few exceptions to restrictive RBC thresholds |
| FRA |  |  | References to specific guidelines |  |
| CHN | Referring to: Single-unit transfusion policy:  Noted that blood resources must be protected and used rationally to avoid unnecessary transfusion. | | | |
| AUT | Referring to: Single-unit transfusion policy:  Noted in text, that a single dose of RBC-concentrate may be sufficient to achieve target values.  Referring to: Transfusion indication for plasma/cryoprecipitate/concentrates/PC/WBC:  Targeted use of procoagulant substances is recommended, based on coagulation status. Citing the ESA guideline (2013). The text advises avoiding inappropriate transfusion of FFP.  For cases of platelet refractoriness, reference is made to „Querschnitts-Leitlinien zur Therapie mit Blutkomponenten und Plasmaderivaten, 4. Auflage“. | | | |
| EU/EC | Advises applying restrictive/symptomatic transfusion thresholds |  |  |  |
| WHO | Referring to: defined RBC-transfusion thresholds [g/dL^-1^]¶  Notes the support of clinical PBM-processes and adherence to guidelines recommending more restrictive transfusion thresholds and their clinical roles | | | |
| ACCP (CHEST) | Recommends a restrictive RBC transfusion strategy over a permissive one for specified indications |  |  |  |

Table S3 Differences in the spotlight - 3. PBM pillar
